# Supplementary material for: Comparative multiomics analysis of cell physiological state after culture in a basket bioreactor
Source: Sci Rep. 2022 Nov 23;12:20161. doi: 10.1038/s41598-022-24687-4 (PMC9686226; doi:10.1038/s41598-022-24687-4)
Supplement: Supplementary file 1 — Supplementary Information 1. [file 41598_2022_24687_MOESM1_ESM.zip › raw data/Transcriptomic raw data/6.Enrichment/2.KEGG/TE1vsTC1/ALL/src/csab04213.html]

csab04213


- csab:103224900

- Down regulated genes

103224900(-1.42998371610984)

- csab:103214788

- Up regulated genes

103214788(1.09310575924899)

- csab:103224900

- Down regulated genes

103224900(-1.42998371610984)

- csab:103240818

- Down regulated genes

103240818(-1.81619424115971)

- csab:103240818

- Down regulated genes

103240818(-1.81619424115971)

- csab:103240818

- Down regulated genes

103240818(-1.81619424115971)

- csab:103223677

- Down regulated genes

103223677(-2.64469587337696)

- csab:103240818

- Down regulated genes

103240818(-1.81619424115971)

Close
